# Changes in musculoskeletal disease activity and patient-reported outcomes in patients with psoriatic arthritis treated with ixekizumab: results from a real-world US cohort

**DOI:** 10.3389/fmed.2023.1184028

**Published:** 2023-06-21

**Authors:** William Tillett, Julie Birt, Cristi Cavanaugh, Yoojin Jung, Aisha Vadhariya, Sarah Ross, Jessica Paulus, Ennio Lubrano

**Affiliations:** ^1^Department of Rheumatology, Royal National Hospital for Rheumatic Disease, Bath, United Kingdom; ^2^Eli Lilly and Company, Indianapolis, IN, United States; ^3^OM1, Inc., Boston, MA, United States; ^4^Academic Rheumatology Unit, Dipartimento Di Medicina e Scienze, Della Salute “Vincenzo Tiberio”, Università Degli Studi del Molise, Campobasso, Italy

**Keywords:** psoriatic arthritis, real world evidence (RWE), musculoskeletal, ixekizumab, patient reported outcomes

## Abstract

**Introduction:**

Ixekizumab has demonstrated efficacy in pivotal trials in patients with psoriatic arthritis (PsA), both those naïve to prior biologic therapy and those with prior inadequate response or intolerance to biologics; however, minimal information is currently available on the effectiveness of ixekizumab in routine clinical practice. The objective of this study was to investigate the clinical effectiveness of ixekizumab for the treatment of PsA over 6- and 12-month follow-up periods in a real-world setting.

**Methods:**

This retrospective cohort study included patients who initiated treatment with ixekizumab from the OM1 PremiOM^TM^ PsA dataset, a dataset of over 50,000 patients with claims and electronic medical record (EMR) data. Changes in musculoskeletal outcomes, such as tender and swollen joint count and patient-reported pain, as well as physician and patient global assessment, as measured using the Clinical Disease Activity Index (CDAI), and Routine Assessment of Patient Index Data 3 (RAPID3) were summarized at 6 and 12 months. The RAPID3, CDAI score, and their individual components were assessed in multivariable regressions adjusting for age, sex, and baseline value. The results were stratified by biologic disease-modifying antirheumatic drug (bDMARD) status (naïve vs. experienced) and monotherapy status (monotherapy vs. combination therapy with conventional synthetic DMARDs). Changes in a 3-item composite score derived from a physician global assessment, patient global assessment, and patient-reported pain score were summarized.

**Results:**

Among the 1,812 patients identified receiving ixekizumab, 84% had prior bDMARD treatment and 82% were monotherapy users. All outcomes improved at 6 and 12 months. For RAPID3, the mean (SD) change at 6 and 12 months was −1.2 (5.5) and −1.2 (5.9), respectively. Patients overall, bDMARD experienced, and monotherapy patients achieved statistically significant mean change in CDAI and all components from baseline to 6 and 12 months in adjusted analyses. Patients experienced an improvement in the 3-item composite score at both time points.

**Conclusion:**

Treatment with ixekizumab was associated with improvements in musculoskeletal disease activity and PROs as assessed by several outcome measures. Future research should assess ixekizumab's clinical effectiveness in the real world across all PsA domains using PsA-specific endpoints.

## Introduction

Psoriatic arthritis (PsA) is a chronic inflammatory arthritis affecting up to 30% of patients with psoriasis (1–4). In the United States (US), the prevalence of PsA ranges from 6 to 25 cases per 10,000 people (5). The clinical presentation of PsA can be heterogenous involving not only the peripheral joints but also the enthesis, axial skeleton, and skin, including the nails.

Therapeutic options for PsA have grown in recent years and are continuing to expand. Currently, available biologic DMARDs (bDMARDs) include tumor necrosis factor (TNF) inhibitors, anti-interleukin (IL)-17, anti-IL-12/23, and anti-IL-23 therapies. Targeted synthetic DMARDs (tsDMARDs) such as Janus kinase (JAK) inhibitors and apremilast, an oral phosphodiesterase 4 inhibitor, are other therapeutic options for patients with PsA (6–8). Given the heterogeneity in PsA manifestations, contemporary treatment guidelines recommend the choice of bDMARD or tsDMARD class be based on the domain(s) of PsA involved in an individualized approach (9).

The varied clinical presentation of PsA has resulted in the development of several PsA-specific composite disease activity indices (10, 11). While some of these composites encompass several PsA domains, others focus more on the musculoskeletal or joint aspects of the condition. There currently is a lack of agreement across the medical community on what the gold standard composite assessment for PsA should be (12). From a real-world perspective in the US, routine rheumatology clinical practice settings often rely on multi-item outcome assessments for PsA patients originally employed in rheumatoid arthritis (RA) such as the Disease Activity Score (DAS28) or the Clinical Disease Activity Index (CDAI), which are commonly incorporated in rheumatology electronic medical record (EMR) systems in the US (13). Such instruments capture the multidimensional aspects of PsA through the use of measures such as the Physician Global Assessment (PhGA) and Patient Global Assessment (PtGA), which have been shown individually to be reliable in PsA (14, 15); however, these composites also include an abbreviated joint count and do not specifically evaluate other domains of PsA. In real-world datasets that do not include multidimensional PsA-specific composite endpoints, nor the assessment of other extra-articular components, physician and patient global assessments, as well as a patient-reported pain, which is also valid and reliable in PsA (16), could be used as alternatives to assess disease activity, as these are often more widely available.

Ixekizumab is a high-affinity monoclonal antibody that selectively targets interleukin (IL)-17A, which was approved by the US Food and Drug Administration (FDA) in December 2017 for patients with active PsA. Ixekizumab demonstrated superior efficacy compared to placebo in two pivotal trials in patients either naïve to prior biologic therapy or patients with prior inadequate response or intolerance to TNF inhibitors, (17, 18) and demonstrated superior efficacy based on a combined joint and skin endpoint (PASI100 and ACR50) compared to adalimumab in a head-to-head clinical study in patients naïve to prior biologic therapy (19). These clinical trials also demonstrated that ixekizumab improves all PsA domains identified in the Outcome Measures in Rheumatology (OMERACT) PsA core set (20). Data from the real world have confirmed ixekizumab's effectiveness in skin symptoms in patients with psoriasis alone and patients with psoriasis and PsA (21, 22). However, few studies to date have assessed the effectiveness of ixekizumab for musculoskeletal symptoms, defined in this study by tender and swollen joint count, patient-reported pain, and physician and patient global assessment, in patients with PsA in a real-world setting.

The objective of this study was to describe the clinical effectiveness of ixekizumab for musculoskeletal symptoms over 6-month and 12-month follow-up periods, including clinician-reported disease activity scores and patient-reported outcome (PRO) measures in a real-world cohort of patients with PsA in the US.

## Materials methods

### Study design and data source

A retrospective design was used to describe the clinical effectiveness of ixekizumab among a cohort of patients with PsA in the US using the OM1 PremiOM^TM^ PsA dataset (OM1, Inc., Boston, MA). This dataset of over 50,000 patients with PsA is derived from deterministically linked, de-identified, individual-level healthcare claims and EMR data. EMR data are derived from several healthcare systems and rheumatologists' EMR provider systems geographically representative of the US population. The EMR data include encounters, medication history and prescription information, laboratory results, PROs, and clinical observations as documented by a rheumatologist. Additional medical and pharmacy claims data containing coding history on inpatient and outpatient encounters from clinics, acute care facilities, or ambulatory surgical centers are linked to the clinical data described above to fill information gaps in patients' clinical care. At the time of analysis, the OM1 PremiOM™ PsA dataset covered the period from 1 January 2013 through July 2021.

### Patient selection

Patients initiating ixekizumab were identified during the timeframe from 1 December 2017 through January 2021. The first observed prescription, fill, or administration from EMR or claims data during this timeframe was defined as the index date. Patients were required to be at least 18 years old on the index date, have a baseline period of at least 12 months on or before the index date, and have at least one diagnosis code for PsA in the baseline period. Patients were excluded if they had at least one diagnosis code for ankylosing spondylitis in the baseline period. All patients were required to have at least 6 months of available follow-up data after the index date. A subset of the study population with ≥12 months of follow-up data was used to assess 12-month outcomes.

Supplementary Figure 1 illustrates the overall study design. The baseline measure was calculated as the closest measure to the index date within 3 months prior to and including the index date. The 6-month follow-up measure was calculated as the closest measure available in the EMR to 6 months post-index date (+/– 3 months). Finally, the 12-month follow-up measure was calculated as the closest measure to 12 months post-index date (+/– 3 months).

### Baseline demographic and clinical characteristics

Patient demographic and clinical characteristics were described using data from the period on or before the index date. When characterizing patients, certain variables such as the number of prior treatments and comorbidities used all available prior history while variables such as domains of PsA and body mass index (BMI) used a 12-month fixed baseline period. The closest measure to the index date within the 3 months was used to assess the baseline values of the clinical outcome measures described in the section below.

### Outcomes

Changes in clinician-assessed outcomes and PROs were assessed from baseline to 6- and 12-months post-index date among patients with available information in the EMR. Clinician-assessed outcomes included: tender joint count (TJC; range 0–28), swollen joint count (SJC; range 0–28), and Physician's Global Assessment (PhGA; range 0–10). In addition, a total Clinical Disease Activity Index score (CDAI; range 0–76) as recorded in the electronic health record at baseline and at the 6- and 12-month post-index date were assessed (23).

PROs included RAPID3 (range 0–30), which is validated in PsA and comprised of the Patient's Global Assessment (PtGA; range 0–10), pain visual analog scale (VAS; range 0–10), and Multidimensional Health Assessment Questionnaire (MDHAQ) Functional Index (FI; range 0–10) (24). A fatigue VAS (range 0–10) was also measured.

### Exploratory outcomes

As an exploratory outcome, measures that have been shown individually to be reliable in PsA, specifically the PhGA (14), PtGA (15), and patient-reported pain VAS (16) were combined to form a PsA-specific composite, where the three 0–10 scales were added together and divided by three for an overall 0–10 total score for the three endpoints (25). Additionally, the PtGA was used to estimate the percentage of patients achieving low disease activity, by using the Minimal Disease Activity (MDA) definition of the PtGA scale (≤2 using a 0–10 VAS) (26).

### Stratification by treatment history and concomitant medications

Changes in clinician-assessed outcomes and PROs were also assessed within strata defined by bDMARD status and concomitant csDMARD therapy status. For bDMARD status, patients were classified as naïve or experienced with experienced patients defined as having had a prescription, fill, or administration of abatacept, adalimumab, certolizumab, etanercept, golimumab, guselkumab, infliximab, risankizumab, secukinumab, or ustekinumab (indication for use not specified in the dataset) during the 12-month baseline period. For concomitant csDMARD therapy status, patients were classified as having initiated ixekizumab as monotherapy or combination therapy, with combination therapy users defined as having a prescription, fill, or administration for auranofin, azathioprine, cyclosporine, leflunomide, methotrexate, or sulfasalazine occurring within 2 months before or 2 months after, and inclusive of, the ixekizumab index date.

### Statistical analysis

Clinician-reported outcomes and PROs as available in the EMR were summarized at baseline and follow-up. The mean [standard deviation (SD)] changes in outcome measures from baseline to 6 and 12 months were summarized descriptively for all patients for each of the components of the outcomes, as well as for the precalculated scores available in the EMR for the total CDAI and RAPID3. Patients who did not have baseline and/or follow-up measures were excluded from the analyses. The number of patients included in each analysis is denoted in the results tables.

A mixed effects linear model was also used to model the changes from baseline to each time point (6 and 12 months) for CDAI and each of its components (TJC, SJC, PtGA, and PhGA) as a function of age, sex, and baseline value for the particular outcome being assessed. The results were also stratified by bDMARD treatment status (naïve vs. experienced) and by concomitant csDMARD therapy status at initiation of ixekizumab (monotherapy vs. combination therapy). The model was not run for particular treatment stratifications and outcome measures if the number of patients in the model was <30 because each model had three covariates (age, sex, and baseline score) and to ensure there were at least 10 patients per covariate in the model.

CDAI was also analyzed categorically by pre-defined disease activity levels. The frequency and percentage of patients achieving remission (CDAI ≤ 2.8), low disease activity (>2.8 and ≤10), moderate disease activity (>10 and ≤22), and high disease activity (>22) were described at baseline and follow-up (23). The frequency and percentage of patients who experienced any level of improvement in disease activity category from baseline to endpoint were reported.

As an exploratory outcome, the combined 3 VAS measure was derived at baseline and follow-up, and the mean (SD) changes from baseline to 6 and 12 months were summarized descriptively. The frequency and percentage of patients reaching a low level of disease activity on the PtGA (≤2) at 6- and 12-month follow-ups were also described. All statistical analyses were performed in SAS (Cary, North Carolina, USA) version 9.4.

## Results

### Baseline characteristics

After applying eligibility criteria to the 51,513 patients in the OM1 PremiOM™ PsA dataset at the time of analysis, 1,812 patients were included in the study population who had initiated ixekizumab with at least 12 months of available data before initiation and at least 6 months of follow-up after initiation. The mean (SD) age was 53.7 (12.2) years, and 61.1% of patients were women. Of patients with known race (78.6% of the cohort), the largest percentage were white (96.5%). All census regions were represented with the majority of patients being from the South (65.3%) (Table 1). Based on medical claims during the 12-month pre-index period, 28% had evidence of enthesitis, 12% synovitis or tenosynovitis, 7% sacroiliitis, and 82% psoriasis. For all patients, the mean (SD) Charlson Comorbidity Index score was 1.3 (1.6), with a high prevalence of other comorbidities (Supplementary Table 1).

**Table 1:** Baseline demographic and clinical characteristics by bDMARD treatment status and csDMARD therapy status for patients with PsA initiating ixekizumab.

		**bDMARD treatment status**		**csDMARD therapy status**		
		**Naïve (*****N** =* **298)**	**Experienced (*****N** =* **1,514)**	**Monotherapy (*****N** =* **1,485)**	**Combination therapy**^1^ **(*****N** =* **327)**	**Total (*****N** =* **1,812)**
**Demographic characteristics**						
Age (years)	Mean (s.d.)	52.7 (13.1)	53.9 (11.9)	53.9 (12.3)	52.9 (11.7)	53.7 (12.2)
	Median (Q1–Q3)	54 (44–62)	55 (46–62)	55 (46–62)	54 (45–61)	55 (46–62)
Sex	Female	191 (64.1%)	917 (60.6%)	909 (61.2%)	199 (60.9%)	1,108 (61.1%)
	Male	107 (35.9%)	597 (39.4%)	576 (38.8%)	128 (39.1%)	704 (38.9%)
Race	Black	4 (1.7%)	20 (1.7%)	20 (1.7%)	4 (1.5%)	24 (1.7%)
	White	221 (94.8%)	1,153 (96.8%)	1,121 (96.4%)	253 (96.9%)	1,374 (96.5%)
	Other	8 (3.4%)	18 (1.5%)	22 (1.9%)	4 (1.5%)	26 (1.8%)
	Unknown	65	323	322	66	388
Insurance	Commercial	140 (75.7%)	698 (74.0%)	692 (74.8%)	146 (71.9%)	838 (74.3%)
	Medicare	35 (18.9%)	178 (18.9%)	175 (18.9%)	38 (18.7%)	213 (18.9%)
	Medicaid	4 (2.2%)	26 (2.8%)	22 (2.4%)	8 (3.9%)	30 (2.7%)
	Other	6 (3.2%)	41 (4.3%)	36 (3.9%)	11 (5.4%)	47 (4.2%)
	Unknown	113	571	560	124	684
Census region	Midwest	25 (8.4%)	145 (9.6%)	132 (8.9%)	38 (11.6%)	170 (9.4%)
	Northeast	43 (14.4%)	287 (19.0%)	274 (18.5%)	56 (17.1%)	330 (18.2%)
	South	206 (69.1%)	976 (64.6%)	963 (64.9%)	219 (67.0%)	1,182 (65.3%)
	West	24 (8.1%)	104 (6.9%)	114 (7.7%)	14 (4.3%)	128 (7.1%)
	Unknown	0	2	2	0	2
**Prior treatment**						
Number of prior biologics	0	298 (100.0%)	0 (0.0%)	245 (16.5%)	53 (16.2%)	298 (16.4%)
	1	0 (0.0%)	522 (34.5%)	424 (28.6%)	98 (30.0%)	522 (28.8%)
	2	0 (0.0%)	436 (28.8%)	370 (24.9%)	66 (20.2%)	436 (24.1%)
	≥3	0 (0.0%)	556 (36.7%)	446 (30.0%)	110 (33.6%)	556 (30.7%)
Number of prior biologicsamong those with at least one prior biologic	N Available	N/A	1,514	1,240	274	1,514
	Mean (s.d.)	N/A	2.3 (1.3)	2.3 (1.3)	2.3 (1.4)	2.3 (1.3)
Prior tsDMARD use	N (%)	90 (30.2%)	625 (41.3%)	600 (40.4%)	115 (35.2%)	715 (39.5%)
Number of prior tsDMARDS among those with at least one prior tsDMARD	N Available	90	625	600	115	715
	Mean (s.d.)	1.1 (0.3)	1.1 (0.4)	1.1 (0.3)	1.2 (0.4)	1.1 (0.3)
csDMARD Combination Therapy^1^	N (%)	53 (17.8%)	274 (18.1%)	0 (0.0%)	327 (100.0%)	327 (18.0%)
**Disease activity measures**						
CDAI	N Available	32	259	237	54	291
	Mean (s.d.)	15.3 (13.9)	15.4 (11.0)	14.3 (10.0)	20.1 (15.0)	15.4 (11.3)
TJC	N Available	60	393	362	91	453
	Mean (s.d.)	3.6 (6.2)	4.0 (6.0)	3.5 (5.4)	5.5 (7.9)	3.9 (6.0)
SJC	N Available	60	394	362	92	454
	Mean (s.d.)	1.9 (3.9)	2.0 (4.0)	1.9 (3.9)	2.5 (4.4)	2.0 (4.0)
PhGA	N Available	55	363	335	83	418
	Mean (s.d.)	2.2 (2.9)	3.2 (2.6)	3.0 (2.7)	3.2 (2.6)	3.0 (2.7)
PtGA	N Available	91	550	514	127	641
	Mean (s.d.)	4.8 (3.1)	5.1 (2.7)	4.9 (2.8)	5.5 (2.8)	5.0 (2.8)
Polyarthritis^2^ among patients with TJC or SJC available	N (%)	17 (28.3%)	148 (37.6%)	122 (33.7%)	43 (46.7%)	165 (36.3%)
**In patients with baseline CDAI (n** = **291)**						
CDAI high disease activity (score >22)	N (%)	–	–	–	–	63 (21.6)
CDAI moderate disease activity (score >10 & ≤22)	N (%)	–	–	–	–	114 (39.2)
CDAI low disease activity (score >2.8 & ≤10)	N (%)	–	–	–	–	86 (29.6)
CDAI remission (score ≤2.8)	N (%)	–	–	–	–	28 (9.6)
RAPID3	N Available	74	404	381	97	478
	Mean (s.d.)	12.9 (7.3)	12.5 (6.8)	12.2 (6.9)	13.8 (7.0)	12.6 (6.9)
Pain VAS	N Available	85	464	438	111	549
	Mean (s.d.)	5.5 (3.1)	5.3 (2.8)	5.2 (2.9)	5.9 (2.7)	5.3 (2.9)
MDHAQ FI	N Available	81	450	426	105	531
	Mean (s.d.)	2.8 (2.3)	2.6 (2.1)	2.6 (2.1)	2.8 (2.2)	2.6 (2.2)
Fatigue VAS	N Available	49	193	183	59	242
	Mean (s.d.)	5.7 (3.2)	5.0 (3.1)	5.2 (3.2)	4.9 (3.0)	5.1 (3.1)

bDMARD, biological disease-modifying anti-rheumatic drug; csDMARD, conventional synthetic biological disease-modifying anti-rheumatic drug; tsDMARD, targeted synthetic disease-modifying anti-rheumatic drug; CDAI, Clinical Disease Activity Index; TJC, tender joint count; SJC, swollen joint count; PtGA, Patient's Global Assessment; PhGA, Physician's Global Assessment; RAPID3, Routine Assessment of Patient Index Data 3; Pain VAS, Pain visual analog scale; MDHAQ FI, Multidimensional Health Assessment Questionnaire Functional Index; Fatigue VAS, Fatigue visual analog scale. ^1^Defined as a prescription, fill, or administration for a csDMARD occurring within 2 months before or 2 months after the index date (inclusive of the index date). ^2^Polyarthritis is defined as >4 joints affected (tender and/or swollen).

Most patients had prior biologic experience (*n* = 1,514, 83.6%) with the mean (SD) number of bDMARDs used during all available prior history of 2.3 (1.3) (Table 1). The most common bDMARDs used immediately before ixekizumab were secukinumab and adalimumab (Table 1). The majority (82%; *n* = 1,485) of patients initiated ixekizumab as monotherapy, with 18% (*n* = 327) initiating ixekizumab in combination with a csDMARD. Demographics were generally similar to the overall population for bDMARD naïve and experienced patients, as well as those initiating ixekizumab as monotherapy or combination therapy (Table 1).

Arthritis with at least one tender or at least one swollen joint was present in 55.4 and 37.7% of patients with a joint count assessment, respectively, with an overall mean (SD) number of tender or swollen joints at the time of initiation of ixekizumab for the total sample of 3.9 (6.0) [(= 453) and 2.0 (4.0) (*n* = 454)], respectively. Of the total sample, 36.3% of patients with a joint count assessment had polyarthritis (defined as >4 joints involved). In patients with PhGA available at baseline (*n* = 418), the mean (SD) was 3.0 (2.7). In patients with a baseline total CDAI score available in the EMR (*n* = 291), 60.8% had moderate to high disease activity at the time of ixekizumab initiation (Table 1). PROs of pain, PtGA, and fatigue demonstrated a moderate burden of disease at baseline from the patient's perspective (Table 1).

### Clinician-assessed disease activity measures

The total CDAI, TJC/SJC, and PhGA individual assessments decreased/improved from baseline to 6 and 12 months, with all endpoints having similar or greater improvement 12 months after initiating ixekizumab compared to 6 months (Supplementary Table 2). In the multivariable analyses adjusting for age, sex, and baseline value, the mean changes in TJC, SJC, PhGA, PtGA, and total CDAI were statistically significant at 6 and 12 months. The mean change in TJC was−1.2 (*p* < 0.0001) at 6 months and −1.3 (*p* = 0.0002) at 12 months. The mean change in SJC was −0.5 (*p* = 0.0009) at 6 months and −0.8 (*p* = 0.0004) at 12 months. The mean change in PhGA and PtGA were −0.9 and −0.7 at 6 months, respectively, and−1.1 and −0.6 at 12 months, respectively (all *p* < 0.0001). The CDAI mean change was −3.5 and −4.3 at 6 and 12 months, respectively (all *p* < 0.0001).

At 6 months, of the patients with non-missing CDAI values (*n* = 168), 40.5% had low disease activity and 11.3% were in remission. At 6 months, 33.3% achieved improvement in any CDAI category, which further increased to 41.3% at 12 months (Table 1).

### Patient-reported outcome measures

RAPID3 and all of its components improved from baseline to 6 and 12 months. The fatigue VAS score decreased at 6 months and further improved at 12 months (Supplementary Table 2).

### Multivariable analysis by bDMARD treatment status and csDMARD therapy status

In the multivariable analyses for bDMARD experienced and bDMARD naïve patients, bDMARD experienced patients achieved a significant mean change in CDAI and all its components from baseline to both 6 and 12 months (Table 2). For bDMARD naïve patients, due to inadequate sample size, the model was not able to be run for the total CDAI or the PhGA at 12 months. Statistically significant mean change was seen for the PhGA at 6 months and the PtGA at both 6 and 12 months (Table 3).

**Table 2:** Multivariable analysis^1^ of change in clinical outcomes for bDMARD-experienced patients with PsA.

**Outcome**	**Time point**	***n***	**Baseline mean**	**Mean change**	**Lower 95% CI**	**Upper 95% CI**	***P*-value**
CDAI	Change from baseline to 6 months	147	16.5	−3.6	−5.0	−2.1	**<0.0001**
	Change from baseline to 12 months	120	16.4	−4.1	−6.1	−2.1	**<0.0001**
TJC	Change from baseline to 6 months	229	4.3	−1.3	−1.9	−0.7	**<0.0001**
	Change from baseline to 12 months	179	4.4	−1.3	−2.0	−0.6	**0.0005**
SJC	Change from baseline to 6 months	229	2.0	−0.5	−0.8	−0.2	**0.0033**
	Change from baseline to 12 months	180	2.2	−0.8	−1.3	−0.3	**0.0011**
PtGA	Change from baseline to 6 months	386	5.1	−0.7	−0.9	−0.5	**<0.0001**
	Change from baseline to 12 months	309	5.0	−0.6	−0.9	−0.3	**0.0001**
PhGA	Change from baseline to 6 months	234	3.2	−0.9	−1.1	−0.6	**<0.0001**
	Change from baseline to 12 months	198	3.4	−1.0	−1.3	−0.7	**<0.0001**

CDAI, Clinical Disease Activity Index; TJC, tender joint count in 28 joints; SJC, swollen joint count in 28 joints; PtGA, Patient's Global Assessment; PhGA, Physician's Global Assessment. The model was adjusted for age, sex, and baseline value of the outcome. ^1^The model was adjusted for age, sex, and baseline value of the outcome. Bold refers to statistically significant (*P* < 0.05).

**Table 3:** Multivariable analysis^1^ of change in clinical outcomes for bDMARD naïve patients with PsA.

**Outcome**	**Time point**	***n***	**Baseline mean**	**Mean change**	**Lower 95% CI**	**Upper 95% CI**	***P*-value**
CDAI	Change from baseline to 6 months	Model not run^2^					
	Change from baseline to 12 months	Model not run^2^					
TJC	Change from baseline to 6 months	37	4.2	−0.9	−2.4	0.5	0.1915
	Change from baseline to 12 months	Model not run^2^					
SJC	Change from baseline to 6 months	37	2.1	−0.7	−1.5	0.1	0.0921
	Change from baseline to 12 months	Model not run^2^					
PtGA	Change from baseline to 6 months	63	5.2	−0.7	−1.3	−0.2	**0.0085**
	Change from baseline to 12 months	46	5.5	−0.8	−1.5	−0.1	**0.0310**
PhGA	Change from baseline to 6 months	39	2.6	−1.0	−1.6	−0.4	**0.0015**
	Change from baseline to 12 months	Model not run^2^					

CDAI, Clinical Disease Activity Index; TJC, tender joint count in 28 joints; SJC, swollen joint count in 28 joints; PtGA, Patient's Global Assessment; PhGA, Physician's Global Assessment. ^1^The model was adjusted for age, sex, and baseline value of the outcome. ^2^Model not run due to inadequate sample size (n < 30). Bold refers to statistically significant (P < 0.05).

For ixekizumab monotherapy users at initiation, scores of total CDAI, TJC/SJC, PhGA, and PtGA significantly improved from baseline to both 6 and 12 months (Table 4).

**Table 4:** Multivariable analysis^1^ of change in clinical outcomes for ixekizumab monotherapy users with PsA.

**Outcome**	**Time point**	***n***	**Baseline mean**	**Mean change**	**Lower 95% CI**	**Upper 95% CI**	***P*-value**
CDAI	Change from baseline to 6 months	131	14.3	−3.6	−5.2	−2.0	**<0.0001**
	Change from baseline to 12 months	105	15.1	−4.9	−7.0	−2.7	**<0.0001**
TJC	Change from baseline to 6 months	208	3.7	−1.3	−1.9	−0.6	**<0.0001**
	Change from baseline to 12 months	157	3.9	−1.4	−2.2	−0.7	**0.0002**
SJC	Change from baseline to 6 months	208	1.8	−0.6	−0.9	−0.2	**0.0013**
	Change from baseline to 12 months	157	2.0	−0.8	−1.3	−0.3	**0.0015**
PtGA	Change from baseline to 6 months	356	5.0	−0.8	−1.0	−0.5	**<0.0001**
	Change from baseline to 12 months	280	5.0	−0.7	−1.0	−0.4	**<0.0001**
PhGA	Change from baseline to 6 months	213	3.1	−0.9	−1.2	−0.7	**<0.0001**
	Change from baseline to 12 months	175	3.1	−1.1	−1.4	−0.8	**<0.0001**

CDAI, Clinical Disease Activity Index; TJC, tender joint count; SJC, swollen joint count; PtGA, Patient's Global Assessment; PhGA, Physician's Global Assessment. ^1^The model was adjusted for age, sex, and baseline value of the outcome. Bold refers to statistically significant (*P* < 0.05).

For combination therapy users at initiation, mean change from baseline was statistically significant for the PhGA at both 6 and 12 months, and for the total CDAI score at 6 months. There were no statistically significant changes at either 6 or 12 months for the TJC, SJC, or PtGA (Table 5).

**Table 5:** Multivariable analysis^1^ of change in clinical outcomes for csDMARD combination therapy users with PsA.

**Outcome**	**Time point**	***n***	**Baseline mean**	**Mean change**	**Lower 95% CI**	**Upper 95% CI**	***P*-value**
CDAI	Change from baseline to 6 months	37	22.8	−3.4	−6.3	−0.4	**0.0252**
	Change from baseline to 12 months	Model not run^2^					
TJC	Change from baseline to 6 months	58	6.5	−1.2	−2.3	0.0	0.0510
	Change from baseline to 12 months	43	6.1	−0.8	−2.2	0.6	0.2661
SJC	Change from baseline to 6 months	58	2.9	−0.4	−1.0	0.3	0.2745
	Change from baseline to 12 months	44	2.9	−0.8	−1.8	0.1	0.0945
PtGA	Change from baseline to 6 months	93	5.5	−0.4	−0.9	0.0	0.0521
	Change from baseline to 12 months	75	5.3	−0.3	−0.9	0.2	0.2650
PhGA	Change from baseline to 6 months	60	3.4	−0.7	−1.2	−0.2	**0.0054**
	Change from baseline to 12 months	50	3.5	−1.1	−1.6	−0.5	**0.0003**

CDAI, Clinical Disease Activity Index; TJC, tender joint count; SJC, swollen joint count; PtGA, Patient's Global Assessment; PhGA, Physician's Global Assessment. ^1^The model was adjusted for age, sex, and baseline value of the outcome. ^2^Model not run due to inadequate sample size (n < 30). Bold refers to statistically significant (*P* < 0.05).

### Exploratory outcomes: 3-item composite (PhGA, PtGA, and pain VAS) and PtGA-low disease activity

Patients experienced an improvement from baseline in the 3-item composite score at both 6 and 12 months with a mean decrease of 0.6 at both time points (Table 6).

**Table 6:** Change in exploratory 3-item composite score (average of the physician's global assessment, patient's global assessment, and pain visual analog scale on a scale of 0–10).

		**Patients with 6-month follow-up score *N =* 189**	**Patients with 12-month follow-up score *N =* 158**
Baseline score	Mean (s.d.)	4.5 (2.4)	4.5 (2.3)
	Median (Q1~Q3)	4.7 (2.7–6.3)	4.7 (2.7–6.3)
Follow-up score	Mean (s.d.)	3.8 (2.1)	3.9 (2.2)
	Median (Q1~Q3)	3.8 (2.2–5.3)	3.8 (2.0–5.7)
Change in score from baseline to follow-up	Mean (s.d.)	−0.6 (1.9)	−0.6 (2.1)
	Median (Q1~Q3)	−0.3 (−1.7–0.3)	−0.3 (−1.7–0.3)

Among patients with PtGA assessments at baseline and follow-up, 26.5% of patients at 6 months and 28.2% of patients at 12 months experienced low disease activity defined as PtGA ≤2.

## Discussion

This study is among the first to provide evidence of the effectiveness of ixekizumab in US patients with PsA from a real-world cohort. Of the 1,812 patients included in the analysis, the majority (84%) were bDMARD experienced with two-thirds of bDMARD-experienced patients having received two or more prior biologic therapies. At baseline, considering the individual outcome assessments, the patient population generally had a low level of disease activity as assessed by clinicians and a moderate level as assessed by patients, which could be related to the extensive prior treatment history. Approximately 64% of the overall population presented with oligoarthritis and 36% with polyarthritis. The composite outcome measures available in the OM1 PremiOM™ PsA dataset were the CDAI and RAPID3. Familiarity with and frequency of use of these assessments in RA has resulted in CDAI and RAPID3 calculators being built into EMR systems used by rheumatologists across the US, further simplifying and proliferating the use of these outcome measures across other disease states. In addition to the pre-calculated total composite scores, individual physician and patient global assessments, as well as patient-reported pain, were used to assess disease activity.

After treatment with ixekizumab, all individual outcomes and total composite scores decreased/improved from baseline to 6 and 12 months, and multivariable analyses demonstrated a statistically significant reduction in disease activity including PtGA, PhGA, TJC, and SJC at both 6 and 12 months. While the absolute change in scores from baseline is relatively small, the baseline and change scores are similar to another longitudinal observational study [PsA Research Consortium (PARC)]. The PARC cohort was used to estimate minimal clinically important improvement across several patient and physician-reported outcomes in a routine clinical practice setting (27). The authors found that all of the measures were able to discriminate between patients who either improved or worsened, but due to the fairly low level of disease activity at baseline, there was minimal room to improve, resulting in the identified clinically meaningful improvement values being smaller compared to what has been identified in RCTs. The mean change in outcome measures we found in our study were within the ranges of the defined clinically relevant changes in the Karmacharya study; however, additional research is needed to determine the optimal minimal clinically relevant levels of improvement when assessed in a real-world practice setting.

Disease activity levels trended down over time with 33% and 41% of patients achieving improvement in any CDAI category at 6 and 12 months, respectively. The percentage of patients who were identified as being in low disease activity or remission increased from 39% at baseline to 52% and 54% at 6 and 12 months, respectively. For bDMARD-experienced patients and ixekizumab monotherapy users, the mean change in total CDAI and its components decreased significantly at both 6 and 12 months when adjusting for age, sex, and baseline value of the measure. While patients with PsA with a history of multiple prior biologics can be more difficult to treat, we saw similar improvements in the clinician and patient-reported assessments after ixekizumab treatment when stratified by biologic naïve and biologic experienced.

We conducted additional exploratory outcome analyses using a 3-item composite of PhGA, PtGA, and pain VAS which was supportive of our primary analyses and also demonstrated an improvement in disease activity with ixekizumab use. This 3-item composite is a variation of a previously established 4-item composite, where the PtGA scale replaces two separate patient assessments of overall joint and skin symptoms. The 4-item composite has been shown to highly correlate with other multi-domain PsA composite endpoints (25). Confirmation of similar correlations with the exploratory 3-item composite reported here is warranted. Low levels of disease activity were achieved by approximately 30% of patients at 6 and 12 months based on the MDA threshold for the PtGA scale. This is similar to the percentage of patients who met the overall MDA criteria in the ixekizumab phase 3 clinical trial in patients with prior TNFi experience (18).

Real-world data on the clinical effectiveness of ixekizumab in patients with PsA is currently limited. Four small studies conducted outside of the US assessed changes in a variety of disease activity scores at 6 and/or 12 months among patients treated with ixekizumab. Chiricozzi et al. (28) assessed a subset of patients with PsA (*n* = 31) from a retrospective observational study of patients with moderate to severe plaque psoriasis. Using the DAS28, they found a mean reduction in score from 4.6 at baseline to 1.4 at 6 months. Berman et al. (29) reported on 23 patients with PsA with previous failure to secukinumab and described statistically significant improvement after 6 and 12 months of ixekizumab treatment as assessed by TJC and several disease activity measures including CDAI, Simplified Disease Activity Index (SDAI), and Disease Activity in Psoriatic Arthritis (DAPSA) score. Darabian et al. (30). reported on data from a chart review on 8 patients with PsA with previous failure to secukinumab. After 12 weeks on ixekizumab, improvement was reported for a majority of patients in TJC and SJC, enthesitis [using the Spondyloarthritis Consortium of Canada (SPARCC) enthesitis score], dactylitis, spondylitis [using the Bath Ankylosing Spondylitis Disease Activity Index (BASDAI)], and psoriasis. Finally, Manfreda et al. (31) reported data from a chart review of 26 patients with PsA and moderate to severe psoriasis and described statistically significant improvement at 6 months in TJC, pain VAS, and DAPSA.

Strengths of our analysis include the assessment of patients with PsA treated with ixekizumab from the largest real-world cohort published to date, and the combined use of both EMR and claims data which enabled a more complete picture of the patient journey. Our data demonstrated improvement in joint and global symptoms in patients treated with ixekizumab assessed by outcome measures determined to be valid and reliable in PsA (i.e., PhGA, PtGA, and RAPID3), and we confirmed improvement in disease activity by several other outcome assessments.

However, there are limitations inherent to retrospective study designs and in the secondary use of data. The diagnosis of PsA was by a rheumatologist clinical assessment, and CASPAR classification criteria were not recorded in the EMR. Missing outcomes data, which could be related to lower and variable frequency of data capture in a real-world setting, compared to clinical trials and outcomes data not captured within the pre-specified outcome windows resulted in lower numbers of patients with data available for some of the outcome measures and inability to run the multivariable models for some of the subgroups. Additionally, we were also unable to assess changes in PsA domain-specific outcomes (e.g., enthesitis and psoriasis) as the quantification of these domains in routine rheumatology practice is limited. Medications ordered (e.g., as documented in the EMR) and prescriptions filled (as documented in the claims data) are proxies for actual use. There are also limitations related to the CDAI, as it is considered an inferior method to fully assess peripheral joint activity in PsA related to the use of the 28-joint count, although the 28-joint count has been demonstrated to correlate with the 68-tender joint count and to be a responsive measure and discriminative for detecting differences between active drug and placebo in a clinical trial setting of patients with PsA with the polyarticular phenotype (32). Despite the limitations, the CDAI has been used as an outcome measure in PsA in clinical trials (33, 34) and has been used in rheumatology registries for PsA (35–37). Nevertheless, the 66/68 joint count is preferred and currently endorsed by OMERACT as part of the PsA Core Outcome set (38).

## Conclusion

In this cohort of patients with PsA and often multiple prior bDMARD failures, improvements in joint disease activity, global assessment, and PROs were observed at 6 months and maintained out to 12 months after initiating treatment with ixekizumab. After adjustment for key patient and prognostic factors, improvements were experienced for outcomes assessed in the total cohort as well as in subgroups by bDMARD experience and ixekizumab monotherapy use. Future research should assess ixekizumab's clinical effectiveness using PsA-specific endpoints across all PsA domains, and additional investigation to define minimal clinically relevant improvement for these outcomes assessed in a real-world population is warranted. The exploratory 3-item (PhGA, PtGA, and pain VAS) composite should be further explored using clinical trials or other observational datasets.

## Data availability statement

The original contributions presented in the study are included in the article/Supplementary material, further inquiries can be directed to the corresponding author.

## Ethics statement

Ethical review and approval was not required for the study on human participants in accordance with the local legislation and institutional requirements. Written informed consent for participation was not required for this study in accordance with the national legislation and the institutional requirements. This article is based on de-identified real-world evidence and does not contain any new studies with human participants or animals performed by any of the authors.

## Author contributions

The study conception and design were performed by JB, AV, SR, CC, JP, and YJ. Material preparation, data collection, and analysis were performed by CC and YJ. Interpretation of data was performed by JB, SR, AV, JP, CC, YJ, WT, and EL. The first draft of the manuscript was written by CC and JP. All authors reviewed, commented on versions of the manuscript, read, and approved the final manuscript.
