# Supplementary figures and images for: Changes in musculoskeletal disease activity and patient-reported outcomes in patients with psoriatic arthritis treated with ixekizumab: results from a real-world US cohort
Source: Front Med (Lausanne). 2023 Jun 21;10:1184028. doi: 10.3389/fmed.2023.1184028 (PMC10322216; doi:10.3389/fmed.2023.1184028)

Supplementary Figure 1. Study Design Scheme

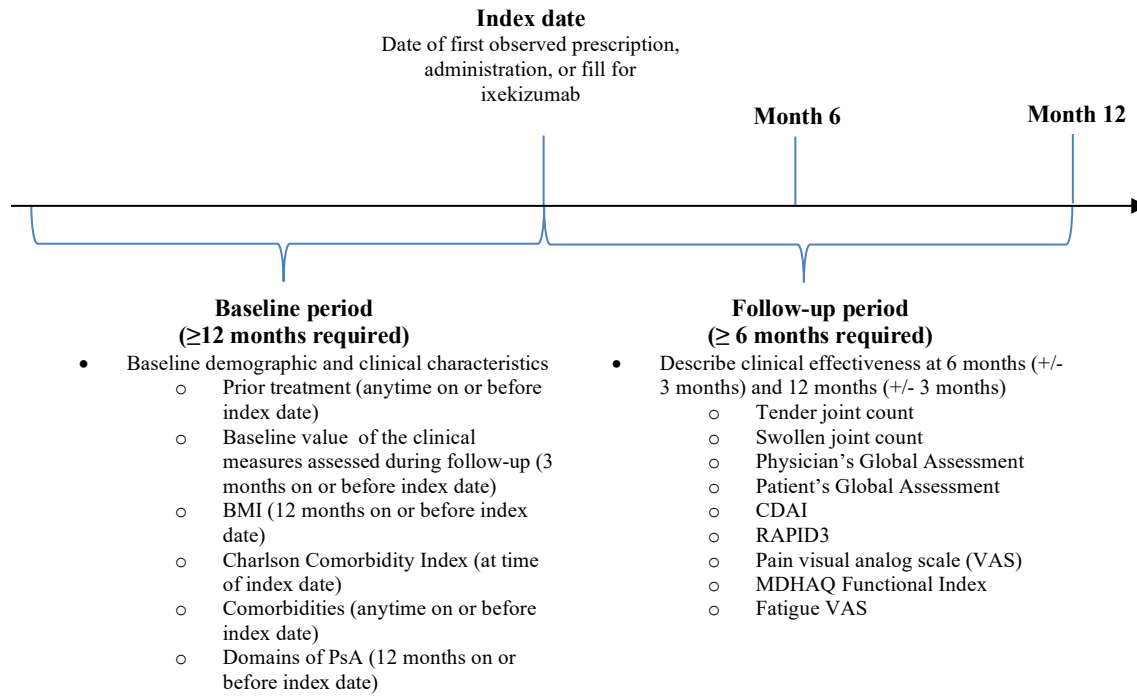

Supplement: Supplementary file 1 [file Image_1.pdf]
