# Supplementary material for: Changes in musculoskeletal disease activity and patient-reported outcomes in patients with psoriatic arthritis treated with ixekizumab: results from a real-world US cohort
Source: Front Med (Lausanne). 2023 Jun 21;10:1184028. doi: 10.3389/fmed.2023.1184028 (PMC10322216; doi:10.3389/fmed.2023.1184028)
Supplement: Supplementary file 2 [file Table_1.docx]

Supplementary Table 1. Additional baseline clinical characteristics by bDMARD treatment status and csDMARD therapy status for patients with PsA initiating ixekizumab

|  | | | | | *bDMARD treatment status* | | | | |  | | *csDMARD therapy status* | | | |  | |
| --- | --- | --- | --- | --- | --- | --- | --- | --- | --- | --- | --- | --- | --- | --- | --- | --- | --- |
|  | | | | | *Naïve (N=298)* | | | *Experienced (N=1,514)* | |  | | *Monotherapy (N=1,485)* | | *Combination Therapy (N=327)* | | *Total (N=1,812)* | |
| **Clinical Characteristics** | |  | |  | | |  | |  | |  | |  | |  | |  |
| Body mass index | | Underweight: <18.5 | | 2 (0.7%) | | | 8 (0.6%) | |  | | 10 (0.7%) | | 0 (0.0%) | | 10 (0.6%) | |  |
|  | | Normal weight: 18.5-24.9 | | 34 (12.5%) | | | 176 (12.2%) | |  | | 172 (12.2%) | | 38 (12.1%) | | 210 (12.2%) | |  |
|  | | Overweight: 25-29.9 | | 85 (31.1%) | | | 370 (25.6%) | |  | | 379 (27.0%) | | 76 (24.2%) | | 455 (26.5%) | |  |
|  | | Obese: >= 30 | | 152 (55.7%) | | | 893 (61.7%) | |  | | 845 (60.1%) | | 200 (63.7%) | | 1,045 (60.8%) | |  |
|  | | Unknown | | 25 | | | 67 | |  | | 79 | | 13 | | 92 | |  |
| Charlson Comorbidity Index | | Mean (s.d.) | | 1.2 (1.4) | | | 1.4 (1.7) | |  | | 1.3 (1.6) | | 1.5 (1.7) | | 1.3 (1.6) | |  |
|  | | Median (Q1-Q3) | | 1 (0-2) | | | 1 (0-2) | |  | | 1 (0-2) | | 1 (0-2) | | 1 (0-2) | |  |
| Comorbidities | |  | |  | | |  | |  | |  | |  | |  | |  |
|  | | Anxiety | | 43 (14.4%) | | | 260 (17.2%) | |  | | 241 (16.2%) | | 62 (19.0%) | | 303 (16.7%) | |  |
|  | | Atherosclerosis and peripheral arterial disease | | 22 (7.4%) | | | 90 (5.9%) | |  | | 86 (5.8%) | | 26 (8.0%) | | 112 (6.2%) | |  |
|  | | Cerebrovascular disease | | 9 (3.0%) | | | 25 (1.7%) | |  | | 27 (1.8%) | | 7 (2.1%) | | 34 (1.9%) | |  |
|  | | Coronary artery disease | | 22 (7.4%) | | | 86 (5.7%) | |  | | 80 (5.4%) | | 28 (8.6%) | | 108 (6.0%) | |  |
|  | | Depression | | 40 (13.4%) | | | 269 (17.8%) | |  | | 243 (16.4%) | | 66 (20.2%) | | 309 (17.1%) | |  |
|  | | Dyslipidemia | | 83 (27.9%) | | | 499 (33.0%) | |  | | 458 (30.8%) | | 124 (37.9%) | | 582 (32.1%) | |  |
|  | | Fibromyalgia | | 36 (12.1%) | | | 241 (15.9%) | |  | | 221 (14.9%) | | 56 (17.1%) | | 277 (15.3%) | |  |
|  | | Hypertension | | 103 (34.6%) | | | 636 (42.0%) | |  | | 599 (40.3%) | | 140 (42.8%) | | 739 (40.8%) | |  |
|  | | Malignant cancer | | 26 (8.7%) | | | 156 (10.3%) | |  | | 149 (10.0%) | | 33 (10.1%) | | 182 (10.0%) | |  |
|  | | Type 2 diabetes | | 49 (16.4%) | | | 263 (17.4%) | |  | | 244 (16.4%) | | 68 (20.8%) | | 312 (17.2%) | |  |
| Domains of PsA | |  | |  | | |  | |  | |  | |  | |  | |  |
|  | | Enthesitis n (%) | | 65 (21.8%) | | | 445 (29.4%) | |  | | 409 (27.5%) | | 101 (30.9%) | | 510 (28.1%) | |  |
|  | | Nail Involvement n (%) | | 15 (5.0%) | | | 66 (4.4%) | |  | | 67 (4.5%) | | 14 (4.3%) | | 81 (4.5%) | |  |
|  | | Psoriasis n (%) | | 233 (78.2%) | | | 1257 (83.0%) | |  | | 1,222 (82.3%) | | 268 (82.0%) | | 1,490 (82.2%) | |  |
|  | | Sacroiliitis n (%) | | 14 (4.7%) | | | 104 (6.9%) | |  | | 91 (6.1%) | | 27 (8.3%) | | 118 (6.5%) | |  |
|  | | Synovitis and Tenosynovitis n (%) | | 27 (9.1%) | | | 184 (12.2%) | |  | | 173 (11.6%) | | 38 (11.6%) | | 211 (11.6%) | |  |
|  |  | |  | | |  | | | | | | | | | | | |
