# Supplementary material for: Changes in musculoskeletal disease activity and patient-reported outcomes in patients with psoriatic arthritis treated with ixekizumab: results from a real-world US cohort
Source: Front Med (Lausanne). 2023 Jun 21;10:1184028. doi: 10.3389/fmed.2023.1184028 (PMC10322216; doi:10.3389/fmed.2023.1184028)
Supplement: Supplementary file 3 [file Table_2.docx]

Supplementary Table 2. Change from baseline in outcomes at 6 months and 12 months

|  | **Baseline** | **6 Months** | **Change from Baseline at 6 Months** | **Baseline** | **12 Months** | **Change from Baseline at 12 Months** |
| --- | --- | --- | --- | --- | --- | --- |
| ***CDAI (n)*** | 168 | 168 | 168 | 133 | 133 | 133 |
| Mean (s.d.) | 16.2 (11.5) | 12.8 (10.6) | -3.4 (10.1) | 16.5 (11.9) | 12.8 (12.2) | -3.7 (11.9) |
| *TJC (n)* | 266 | 266 | 266 | 200 | 200 | 200 |
| Mean (s.d.) | 4.3 (6.3) | 3.1 (5.7) | -1.2 (5.0) | 4.4 (6.5) | 3.2 (5.7) | -1.2 (5.5) |
| *SJC (n)* | 266 | 266 | 266 | 201 | 201 | 201 |
| Mean (s.d.) | 2.0 (4.0) | 1.5 (3.2) | -0.5 (3.1) | 2.2 (4.3) | 1.4 (3.4) | -0.8 (4.2) |
| *PtGA (n)* | 449 | 449 | 449 | 355 | 355 | 355 |
| Mean (s.d.) | 5.1 (2.7) | 4.4 (2.7) | -0.6 (2.5) | 5.1 (2.7) | 4.5 (2.8) | -0.6 (2.7) |
| *PhGA (n)* | 273 | 273 | 273 | 225 | 225 | 225 |
| Mean (s.d.) | 3.1 (2.7) | 2.3 (2.3) | -0.8 (2.4) | 3.2 (2.7) | 2.2 (2.3) | -1.0 (2.5) |
| ***RAPID3 (n)*** | 303 | 303 | 303 | 232 | 232 | 232 |
| Mean (s.d.) | 13.0 (6.8) | 11.7 (6.9) | -1.2 (5.5) | 13.1 (6.9) | 11.8 (7.0) | -1.2 (5.9) |
| *Pain VAS (n)* | 356 | 356 | 356 | 270 | 270 | 270 |
| Mean (s.d.) | 5.4 (2.8) | 4.8 (2.8) | -0.6 (2.6) | 5.4 (2.8) | 4.9 (2.8) | -0.5 (2.8) |
| *MDHAQ FI (n)* | 347 | 347 | 347 | 261 | 261 | 261 |
| Mean (s.d.) | 2.7 (2.1) | 2.5 (2.1) | -0.2 (1.5) | 2.8 (2.2) | 2.5 (2.1) | -0.3 (1.6) |
| ***Fatigue VAS (n)*** | 179 | 179 | 179 | 125 | 125 | 125 |
| Mean (s.d.) | 5.0 (3.2) | 4.8 (3.2) | -0.2 (2.7) | 5.3 (3.2) | 4.9 (3.3) | -0.4 (2.7) |

CDAI = Clinical Disease Activity Index; TJC = tender joint count; SJC = swollen joint count; PtGA = Patient’s Global Assessment; PhGA = Physician’s Global Assessment; RAPID3 = Routine Assessment of Patient Index Data 3; Pain VAS = Pain visual analog scale; MDHAQ FI *=* Multidimensional Health Assessment Questionnaire Functional Index*;* Fatigue VAS = Fatigue visual analog scale
